# Lack of Trehalose Accelerates H_2_O_2_-Induced *Candida albicans* Apoptosis through Regulating Ca^2+^ Signaling Pathway and Caspase Activity

**DOI:** 10.1371/journal.pone.0015808

**Published:** 2011-01-05

**Authors:** Hui Lu, ZhenYu Zhu, LingLing Dong, XinMing Jia, XuanRong Sun, Lan Yan, YiFeng Chai, YuanYing Jiang, YingYing Cao

**Affiliations:** School of Pharmacy, Second Military Medical University, Shanghai, People's Republic of China; INSERM U1016, Institut Cochin, France

## Abstract

Trehalose is a non-reducing disaccharide and can be accumulated in response to heat or oxidative stresses in *Candida albicans*. Here we showed that a *C. albicans tps1Δ* mutant, which is deficient in trehalose synthesis, exhibited increased apoptosis rate upon H_2_O_2_ treatment together with an increase of intracellular Ca^2+^ level and caspase activity. When the intracellular Ca^2+^ level was stimulated by adding CaCl_2_ or A23187, both the apoptosis rate and caspase activity were increased. In contrast, the presence of two calcium chelators, EGTA and BAPTA, could attenuate these effects. Moreover, we investigated the role of Ca^2+^ pathway in *C. albicans* apoptosis and found that both calcineurin and the calcineurin-dependent transcription factor, Crz1p, mutants showed decreased apoptosis and caspase activity upon H_2_O_2_ treatment compared to the wild-type cells. Expression of *CaMCA1*, the only gene found encoding a *C. albicans* metacaspase, in calcineurin-deleted or Crz1p-deleted cells restored the cell sensitivity to H_2_O_2_. Our results suggest that Ca^2+^ and its downstream calcineurin/Crz1p/*CaMCA1* pathway are involved in H_2_O_2_ -induced *C. albicans* apoptosis. Inhibition of this pathway might be the mechanism for the protective role of trehalose in *C. albicans*.

## Introduction

*Candida albicans* is the most important human fungal pathogen, causing various diseases from superficial mucosal infections to life-threatening systemic disorders [1]–[3]. The number of clinical *C. albicans* infections worldwide has risen considerably in recent years, and the incidence of resistance to traditional antifungal therapies is also rising. Many existing antifungal therapies have unfortunate clinical side effects; therefore, strategies are needed to identify new targets for antifungal therapy.

In the past few years, it became evident that apoptosis might occur not only in multicellular, but also in unicellular organisms, such as fungi. The induction of cell apoptosis is considered as a new and promising strategy for antifungal therapy. It has been reported that *Saccharomyces cerevisiae* dies in an apoptotic manner in response to weak acid stress, oxidative stress, salt stress, and UV irradiation [4]–[7]. Ultrastructural and biochemical changes that are characteristic of apoptosis have also been reported in pathogenic fungi. *C. albicans* can be triggered to undergo an apoptotic cell death response when exposed to environmental stress such as H_2_O_2_, amphotericin B (AmB) or intracellular acidification. However, the mechanism of *C. albicans* apoptosis has not been fully revealed. Ras–cAMP–PKA was found to be involved in the apoptosis of *C. albicans*. Mutations that blocked Ras–cAMP–PKA signaling (*ras1Δ, cdc35Δ, tpk1Δ, and tpk2Δ*) suppressed or delayed the apoptotic response, whereas mutations that stimulated signaling (*RAS1^val13^* and *pde2Δ*) accelerated the rate of entry into apoptosis [8]–[10]. We recently found that *CaMCA1*, a homologue of *Saccharomyces cerevisiae* metacaspase *YCA1*, was involved in oxidative stress-induced apoptosis in *C. albicans* [11].

Trehalose, a non-reducing disaccharide, plays diverse roles, from energy source to stress protectant, and this sugar is found in bacteria, fungi, plants, and invertebrates but not in mammals [12]. In yeast, trehalose acts both as a main reserve of carbohydrates and as a cellular protector against a variety of nutritional and/or environmental stress challenges (oxidative, heat shock, osmotic and/or saline stress, xenobiotics etc.), increasing cell resistance to such insults [13]. The mechanism of trehalose protection is an active area of research that includes studies of the interaction of sugars with plasma membranes, the effects on cell osmotic responses, and the unique physicochemical properties of trehalose [14]. In yeast, trehalose is synthesized by a large enzyme complex comprising the two catalytic activities of trehalose biosynthesis. Trehalose-6-phosphate (Tre6P) synthase, encoded by *TPS1*, synthesizes Tre6P from glucose-6-phosphate and UDP-glucose. Tre6P is then hydrolyzed into trehalose by Tre6P phosphatase, encoded by *TPS2* [15], [16]. In *C. albicans, tps1/tps1* mutants are defective not only for Tre6P synthesis but also for growth on glucose or related rapidly fermented sugars and virulence [17], [18]. Previous work on *C. albicans* pointed to a specific role of trehalose in cellular protection against oxidative stress. A *tps1/tps1* mutant was shown to be deficient in trehalose synthesis and was extremely sensitive to H_2_O_2_ exposure [19]. However, the underlying mechanism by which trehalose protects *C. albicans* from the injuries remains undefined.

Ca^2+^ is an important second messenger in developmental and stress signaling pathways. In fungi, Ca^2+^ is responsible for the regulation of several processes, including cation homeostasis, morphogenesis, virulence traits, and antifungal drug resistance [20]–[23]. A rise in cytoplasmic Ca^2+^ has been found to be responsible for pheromone-induced *S. cerevisiae* apoptosis [24]. Fungicidal activity of amiodarone is also tightly coupled to calcium influx [25]. A rise in cytosolic calcium activates the calcium-dependent signaling pathway via the phosphatase, calcineurin (consisting of a catalytic subunit A encoded by *CMP1* and a regulatory subunit B encoded by *CNB1*) and the calcineurin-dependent transcription factor, Crz1p. In *C. albicans*, Ca^2+^ and its downstream calcineurin/Crz1p pathway are involved in azole resistance, cell morphogenesis and virulence [26]–[29].

In this study, we show that lack of trehalose can accelerate H_2_O_2_ -induced *C. albicans* apoptosis. Furthermore, this is linked to an increase of Ca^2+^ concentration and caspase activity. Addition or depletion of Ca^2+^ affected the cell death and caspase activity. Moreover, we investigated the role of Ca^2+^ signaling in *C. albicans* apoptosis, and found that both calcineurin-deleted and Crz1p-deleted cells showed decreased cell death and caspase activity compared to the wild-type cells. Expression of *CaMCA1* in calcineurin-deleted or Crz1p-deleted cells restored the sensitivity to H_2_O_2_.

## Results

### Lack of Trehalose Accelerates H_2_O_2_-induced Apoptosis

**Figure 1:**
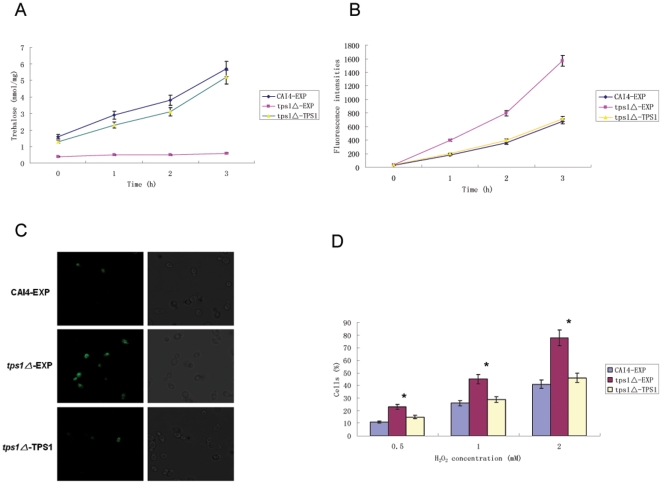
Effects of trehalose accumulation on H_2_O_2_-induced apoptosis and ROS production. (A) The wild-type (CAI4-EXP), *tps1△*-EXP and *tps1△-*TPS1 cells were exposed to 1 mM H_2_O_2_ for up to 3 hours. At the indicated times, aliquots of cells were taken to measure trehalose content. (B) The cells were exposed to 1 mM H_2_O_2_. At the indicated times, aliquots of cells were taken to measure the intracellular ROS by POLARstar Galaxy with excitation at 485 nm and emission at 520 nm. (C) DNA damage of the cells after treatment with 1 mM H_2_O_2_ for 3 hours revealed by the TUNEL assay under a fluorescence microscope. (D) Percentage of cells that were classified as apoptotic by TUNEL assay after treatment with indicated concentrations of H_2_O_2_ for 3 hours using a BD FACS Calibur flow cytometer with excitation and emission wavelength settings at 488 and 520 nm, respectively. These data were mean values ± S.D. from three independent experiments. * indicates P<0.01 compared with values from the control CAI4-EXP cells.

In *C. albicans*, *TPS1* encodes trehalose-6-phosphate (Tre6P) synthase that is required for trehalose synthesis. A *tps1Δ* mutant is deficient in trehalose accumulation. The impact of *TPS1* mutation on trehalose accumulation is shown in Fig. 1A. Trehalose accumulation was increased in wild-type cells after 1 to 3 hours exposure to 1 mM H_2_O_2_. This increase did not appear in *tps1Δ* mutant.

Since it has been reported that H_2_O_2_ can induce apoptosis in *C. albicans* and reactive oxygen species (ROS) is an indicator of apoptosis [9], [22], we examined ROS generation of the cells with the fluorescent dye DCFH-DA. An increase of intracellular ROS level was observed in both *tps1△* mutant and wild-type cells upon H_2_O_2_ treatment. However, this increase was even stronger in *tps1△* mutant (Fig. 1B). Consistent with this, the *tps1△* mutant showed a higher percentage of cells demonstrating ROS accumulation than the wild-type cells (Table 1).

**Table 1:** Percentages of cells demonstrating ROS accumulation after exposure to 1 mM H_2_O_2_ for the indicated time.

Group	1 hour	2 hours	3 hours
CAI4-EXP	7±1	15±2	41±4
*tps1△*-EXP	17±2	48±6*	78±6*
*tps1△-*TPS1	8±1	17±2	34±4

*indicated P<0.01 compared with values from the CAI4-EXP cells.

To ascertain the role of trehalose in *C. albicans* apoptosis, we compared the apoptosis rate between the wild-type cells and *tps1Δ* mutant when exposed to different concentrations of H_2_O_2_. As shown in Fig. 1C, upon H_2_O_2_ treatment, the apoptosis rate of *tps1Δ* mutant was higher than wild-type cells. After 3 hours treatment with 2 mM H_2_O_2_, 78% of the *tps1Δ* mutant cells were apoptotic, while the apoptosis rate of the wild-type cells was 47%.

### Lack of Trehalose Enhances Ca^2+^ Elevation And Caspase Activity

**Figure 2:**
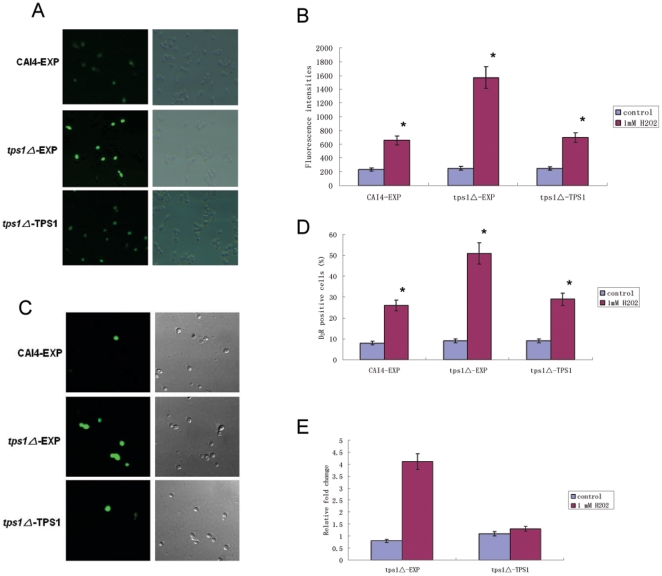
Effects of trehalose accumulation on H_2_O_2_-induced Ca^2+^ elevation and caspase activity. The wild-type (CAI4-EXP), *tps1△*-EXP and *tps1△-*TPS1 cells were exposed to 1 mM H_2_O_2_ for 3 hours and stained with Fluo-3/AM. Ca^2+^ levels were determined by observing the fluorescence using a fluorescence microscope (A) or the POLARstar Galaxy (B). The caspase activity of the cells treated with 1 mM H_2_O_2_ for 3 hours was determined by staining the cells with D_2_R and counting under a fluorescence microscope (C, D). Transcription levels of *CaMCA1* in response to 1 mM H_2_O_2_ for 3 hours determined by real-time RT-PCR. The mRNA levels were normalized on the basis of their *ACT1* levels. Gene expression was indicated as the fold increase of *tps1△*-EXP and *tps1△-*TPS1 cells relative to that of the wild-type (CAI4-EXP) strain (E). These data were mean values ± S.D. from three independent experiments. * indicates P<0.01 compared with values from the control CAI4-EXP cells.

In *S. cerevisiae*, elevation of intracellular Ca^2+^ can lead to cell death [25]. We determined the intracellular Ca^2+^ upon H_2_O_2_ treatment using a fluorescent calcium indicator Fluo-3/AM. In the absence of H_2_O_2_, the intracellular levels of Ca^2+^ in both the *tps1Δ* mutant and wild-type cells were rather low and almost undetectable. After treatment with 1 mM H_2_O_2_ for 3 hours, both of the groups showed obvious elevation of intracellular Ca^2+^, while the *tps1Δ* mutant cells showed a higher level of Ca^2+^ than the wild-type cells (Fig. 2A, 2B).

Since we previously found that the caspase activity was increased in *C. albicans* apoptosis [11], here we investigated the caspase activity by staining the cells with D_2_R, a nonfluorescent substrate, which is cleaved to green fluorescent monosubstituted rhodamine 110 and free rhodamine [10], [11], [30]. As shown in Fig. 2C and 2D, after treatment with 1 mM H_2_O_2_ for 3 hours, the cell number stainable by D_2_R in the wild-type cells was 26%, while that in the *tps1Δ* mutant was 51%. Furthermore, the transcript levels of *CaMCA1*, which is responsible for caspase activity in *C. albicans*, were investigated by real time RT-PCR. As shown in Fig. 2E, in the absence of H_2_O_2_, there was no significant difference in the transcript level of *CaMCA1* between the *tps1Δ* mutant and wild-type cells. However, a 4 fold increase of *CaMCA1* transcript level was recorded in the *tps1Δ* mutant compared to that in the wild-type cells when exposed to 1 mM H_2_O_2_ for 3 hours.

### Adding or Depleting Ca^2+^ Affected Apoptosis and Caspase Activity

**Figure 3:**
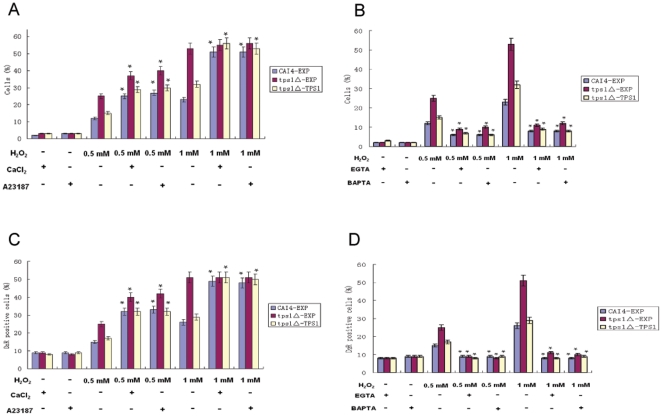
Effects of adding or depleting Ca^2+^ on H_2_O_2_-induced apoptosis and caspase activity. (A, B) The wild-type (CAI4-EXP), *tps1△*-EXP and *tps1△-*TPS1 cells were exposed to 0.5 mM or 1 mM H_2_O_2_ for 3 hours in the absence or presence of CaCl_2_ (0.5 mM), A23187 (0.5 µM), EGTA (1 mM), BAPTA (1 µM). Percentage of cells that were classified as apoptotic by TUNEL assay was shown. (C, D) Caspase activity determined by staining the cells with D_2_R. These data were mean values ± S.D. from three independent experiments. * indicates P<0.01 compared with values from the cells treated with the same concentrations of H_2_O_2_ only.

Since the intracellular Ca^2+^ level could be increased by H_2_O_2_, especially in the *tps1Δ* mutant, we hypothesized that Ca^2+^ signaling might regulate *C. albicans* apoptosis, and the higher sensitivity of *tps1Δ* mutant to H_2_O_2_ might be due to its higher intracellular Ca^2+^ level. As shown in Fig. 3A, when we stimulated the intracellular Ca^2+^ level by adding CaCl_2_ (0.5 mM), the apoptosis rate increased in both the *tps1△* mutant and wild-type cells. Similar effects were observed when A23187 (0.5 µM), a calcium ionophore, was added. CaCl_2_ and A23187 themselves at the concentrations tested had no effects on *C. albicans* growth. In addition, the presence of both CaCl_2_ and A23187 resulted in an increased caspase activity in both the *tps1△* mutant and wild-type cells (Fig. 3C).

Furthermore, we tested the effect of depleting Ca^2+^. As shown in Figure 3B, the presence of EGTA (1 mM), an extracellular calcium chelator, attenuated the H_2_O_2_-induced apoptosis in both *tps1Δ* mutant and wild-type cells, accompanied by the decrease of caspase activity (Fig. 3D). Similarly, when BAPTA (1 µM), an intracellular calcium chelator, was added, both the apoptosis rate and caspase activity in the two strains were decreased.

### Deletion of Calcineurin or Crz1p Leads to a Decrease in Apoptosis and Caspase Activity

**Figure 4:**
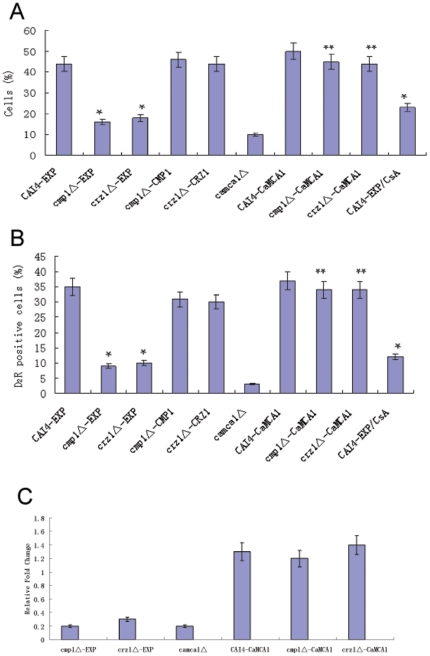
Effects of *CMP1* deletion, *CRZ1* deletion or expression of *CaMCA1* on H_2_O_2_-induced apoptosis and caspase activity. The wild-type (CAI4-EXP), *cmp1Δ*-EXP, *crz1Δ*-EXP, *cmp1Δ-*CMP1, *crz1Δ*-CRZ1 *camca1Δ*, CAI4-CaMCA1, *cmp1Δ-*CaMCA1 and *crz1Δ*-CaMCA1 cells were exposed to 2 mM H_2_O_2_ for 3 hours. In another experiment, the wild-type cells were exposed to 2 mM H_2_O_2_ for 3 hours in the presence of cyclosporin A (0.08 µM). (A) Percentage of cells that were classified as apoptotic by TUNEL assay was shown. (B) The caspase activity was determined by staining the cells with D_2_R. (C) Transcription level of *CaMCA1* in response to 2 mM H_2_O_2_ for 3 hours was determined by real time RT-PCR. The mRNA levels were normalized on the basis of their *ACT1* levels. Gene expression is indicated as the fold increase of the mutant and *CaMCA1*-introduced cells relative to that of the wild-type cells. The data are mean values ± S.D. from three independent experiments. * indicates P<0.01 compared with values of CAI4-EXP treated with H_2_O_2_ only. ** indicates P<0.01 compared with values of parental cells without *CaMCA1*.

In *C. albicans*, calcineurin and Crz1p are two major proteins involved in Ca^2+^ signaling and play an important role in antifungal tolerance, cell morphogenesis and virulence [20], [21], [26]. So it is possible that the effects of Ca^2+^ on cell death are mediated by calcineurin and its downstream target Crz1p. To test this hypothesis, we examined the viability of calcineurin and Crz1p mutants [27] upon H_2_O_2_ treatment. After 3 hours treatment with 2 mM H_2_O_2_, 52% of wild-type cells were apoptotic while the apoptosis rates of *cmp1Δ* and *crz1Δ* mutants were 19% and 25%, respectively. In the *cmp1Δ-*CMP1 and *crz1Δ*-CRZ1 cells which contain reintroduced *CMP1* and *CRZ1* gene, the apoptosis rate was similar to the wild-type cells (Fig. 4A). As expected, the caspase activities in both the *cmp1Δ* and *crz1Δ* mutants were lower than that in wild-type cells (Fig. 4B). Consistent with this, the transcription levels of *CaMCA1* in *cmp1Δ* and *crz1Δ* mutants were much lower than that in the wild-type cells (Fig. 4C). The potential role of calcineurin in H_2_O_2_-induced apoptosis was further examined using the calcineurin inhibitor cyclosporin A. Upon H_2_O_2_ treatment, the wild type cells showed lower apoptosis rates and caspase activity in the presence of 0.08 µM cyclosporin A as compared to the absence of this compound (Fig. 4A, 4B).

### Expression of *CaMCA1* in Calcineurin-deleted and Crz1p-deleted Cells Restored the Sensitivities to H_2_O_2_

Since the caspase activity was decreased in *cmp1Δ* and *crz1Δ* mutants upon H_2_O_2_ exposure, we introduced *CaMCA1* into the *cmp1Δ* and *crz1Δ* mutants and assessed the phenotype. Upon H_2_O_2_ treatment, the apoptosis rates (Fig. 4A) and caspase activities (Fig. 4B) of the *CaMCA1*-introduced cells were much higher than the *cmp1Δ* and *crz1Δ* mutants. Consistent with this, the transcription levels of *CaMCA1* in *cmp1Δ* and *crz1Δ* mutants were lower than that in the wild-type cells, while the transcription levels of *CaMCA1* in the *CaMCA1*-introduced cells were similar to that in the wild-type cells (Fig. 4C). In addition, the apoptosis rates and caspase activities of the *camca1Δ* mutant were lower than the wild-type cells. These data indicated that *CaMCA1* could restore the decreased apoptosis and caspase activities of calcineurin-deleted and Crz1p-deleted cells.

## Discussion

In yeasts, trehalose acts both as a main reserve of carbohydrates and as a cellular protector against a variety of nutritional and/or environmental stress challenges, increasing cell resistance to such injuries. Trehalose accumulation in *C. albicans* has been described as a defense mechanism against oxidative stress. A trehalose-deficient *tps*1Δ mutant is highly sensitive to H_2_O_2_ and prone to undergo phagocytic digestion [31]. However, the mechanism by which trehalose protects *C. albicans* from injuries remains unclear. Since apoptosis is now considered as one of the important ways of *C. albicans* death, we assessed the role of trehalose in H_2_O_2_-induced apoptosis using a *tps1△* mutant. According to our result, lack of trehalose could accelerate H_2_O_2_ -induced apoptosis which was accompanied by an increase of ROS, an apoptosis indicator. This result revealed a mechanism for the protective role of trehalose in *C. albicans*. Similar results were reported by other researchers. Liu *et al.* found that trehalose could inhibit the phagocytosis of refrigerated platelets *in vitro* via preventing apoptosis [32]. Also, trehalose has been found to protect against ocular surface disorders in experimental murine dry eye through suppression of apoptosis [33].

Our detailed studies on the protective effect of trehalose revealed a role of Ca^2+^ signals in *C. albicans* apoptosis. We observed that there was an increase of intracellular Ca^2+^ level in both the *tps1△* mutant and wild-type cells upon H_2_O_2_ treatment. However, this increase was much stronger in *tps1△* mutant, which was consistent with the higher apoptosis rate induced in this strain. When we stimulated the intracellular Ca^2+^ level by adding CaCl_2_ or A23187, the apoptosis rates in both the *tps1△* mutant and wild-type cells were increased. In contrast, when Ca^2+^ was depleted by adding EGTA or BAPTA, the apoptosis rates in both the *tps1△* mutant and wild-type cells were decreased. These results indicated that apoptosis could be induced in *C. albicans* through increasing intracellular Ca^2+^ level.

The role of Ca^2+^ in *C. albicans* apoptosis was further examined by the experiments with *CMP1* and *CRZ1*, two genes involved in Ca^2+^ signaling. We found that *cmp1Δ* and *crz1Δ* mutants showed attenuated apoptosis upon H_2_O_2_ treatment, similar to the effect of depleting Ca^2+^ in wild-type cells. Consistent with this result, addition of cyclosporin A, a calcineurin inhibitor, could also attenuate apoptosis. Taken together, Ca^2+^ and its downstream calcineurin/Crz1p pathway are involved in H_2_O_2_ -induced *C. albicans* apoptosis.

**Figure 5:**
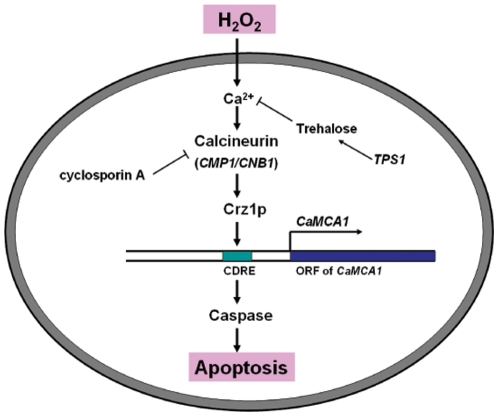
A model for the role of trehalose in the regulation of H_2_O_2_-induced apoptosis in *C. albicans*. When *C. albicans* is exposed to H_2_O_2_, the intracellular Ca^2+^ is increased and its downstream calcineurin/Crz1p pathway is activated. The calcineurin inhibitor cyclosporin A can block this pathway. Crz1p might up-regulate the expression of *CaMCA1* through binding to the CDRE (calcineurin-dependent responsive element) in the promoter of *CaMCA1.* The increased expression of *CaMCA1* results in the increased caspase activity and thus apoptosis occurs. *tps1△* mutation results in the lack of trehalose accumulation thus accelerates *C. albicans* apoptosis.

In mammals, apoptosis can be directed by the activation caspases, which cleave specific substrates and trigger cell death. In the past few years, it has become evident that caspases might exist not only in multicellular, but also in unicellular organisms, such as fungi. In *S. cerevisiae*, *YCA1* encodes a single metacaspase, which has caspase activity. *YCA1* is involved in the apoptosis of yeast cells exposed to different environmental stresses, such as H_2_O_2_, acetic acid, sodium chloride, heat shock, and hyperosmosis [34]–[36]. In plants, metacaspases have been associated with Norway spruce apoptosis during embryogenesis and tomato plant apoptosis induced by fungal infection [37]–[39]. Using yeast as a heterologous system for apoptosis evaluation, the metacaspases *AtMCP1b* and *AtMCP2b* from the plant *Arabidopsis thaliana* were also found to be involved in apoptosis induced by H_2_O_2_ [40]. We recently found that H_2_O_2_-induced *C. albicans* apoptosis was accompanied with caspase activity, which was encoded by *CaMCA1* [11]. In this study, we found that, upon H_2_O_2_ treatment, the caspase activities in *tps1△* mutant were much higher than those in wild-type cells, similar to the phenomena of intracellular Ca^2+^ levels. The positive relation between Ca^2+^ level and caspase activity was proved by adding or depleting Ca^2+^. Moreover, both calcineurin-deleted and Crz1p-deleted cells showed lower caspase activity compared to the wild-type cells, indicating that *CaMCA1* might be a downstream gene which is blocked in calcineurin-deleted or Crz1p-deleted cells (Fig. 5). As expected, when extraneous *CaMCA1* was introduced into these cells, the caspase activity and cell sensitivity to H_2_O_2_ were resumed. Previous studies showed that *C. albicans CaMCA1* could be activated by Ca^2+^ and regulated by calcineurin and Crz1p. Moreover, CDRE (calcineurin-dependent responsive element) was found in the promoter of *CaMCA1* [26]. Based on these results, we conclude that *CaMCA1* is likely to be one of the downstream genes influenced by the Ca^2+^ signaling and involved with the protective role of trehalose against H_2_O_2_-induced apoptosis.

## Materials and Methods

### Media and Compounds

Yeast media used were YPD (1% yeast extract, 2% peptone, and 2% glucose) and SD [0.67% (w/v) Difco yeast nitrogen base without amino acids]. SD medium was supplemented with a complete synthetic mix containing all the amino acids and bases. For prototrophic selection of yeast, the relevant drop-out mixes were used. Because the capacity of the trehalose-deficient mutant *tps1*/*tps1* to grow on exogenous glucose and fructose as carbon source is seriously compromised, some experiments were carried out in YPgal medium (1% yeast extract, 2% peptone, and 2% galactose) or SDgal [0.67% (w/v) Difco yeast nitrogen base without amino acids, 2% galactose]. *Escherichia coli* strain DH5α and LB (0.5% yeast extract, 1% peptone, and 1% NaCl) medium were used for transformation and plasmid DNA preparation. Fluo-3/AM, CaCl_2_, A23187, BAPTA, EGTA, cyclosporin A (Sigma, U.S.A.) were dissolved in either medium or dimethyl sulfoxide (DMSO) and then diluted to the appropriate working concentration.

### Plasmids and Strain Construction

The strains (Table 2) were cultivated at 30°C under constant shaking (200 rpm) or incubation. To reintroduce *TPS1* to *tps1Δ* mutant, the ORF of *TPS1* was amplified (using upstream primer 5′ ggatccatggttcaaggaaaagtc 3′ and downstream primer 5′ ctgcagctagtccctcaaactcttttg 3′) with Pyrobest DNA polymerase (TaKaRa Biotechnology, Dalian, P.R. China). After being purified, the BamHI-PstI digested PCR fragment was cloned into the integrative expression vector pCaEXP (Table 3) to generate the recombinant plasmid pCaEXP-TPS1 [41]. After sequencing, pCaEXP-TPS1 was linearized and used to transform *tps1Δ* cells, and selected on SD medium lacking uridine, methionine and cysteine. As controls, the empty plasmid pCaEXP was transformed into CAI4 and *tps1Δ* cell to produce CAI4-EXP and *tps1Δ*-EXP, respectively. The same expression vector and transformation method were used for reintroducing *CMP1* (using upstream primer 5′ ggatccatgtcaggaaatactgttcaa 3′ and downstream primer 5′ ctgcagttaactttgagataatcttct 3′) and *CRZ1* (using upstream primer 5′ ggatccatgtctaacaatcctcatccc 3′ and downstream primer 5′ ctgcagctaagtaatttcaacaccact 3′) genes to their corresponding mutants, and introducing *CaMCA1* (using upstream primer 5′ ggatccatgtttccaggacaaggtag 3′ and downstream primer 5′ ctgcagttaaaaaataaattgcaagtt 3′) to *cmp1Δ* and *crz1Δ* mutants and CAI4. The expression of *TPS1*, *CMP1*, *CRZ1* and *CaMCA1* in their host cells was confirmed by real time RT-PCR (data not shown).

**Table 2:** *C. albicans* strains used in this study.

Strain	Parent	Genotype	Reference
*CAI4*	*CAF2-1*	*ura3△::immm434/ura3△::immm434*	Fonzi et al., 1993
*CAI4-EXP*	*CAI4*	*ura3△::immm434/ura3△::immm434::URA3*	This study
*cmp1Δ(DSY2091)*	*CAF4-2*	*cmp1△::hisG/cmp1△::hisG-URA3-hisG*	Karababa et al., 2006
*cmp1Δu*	*cnaΔ*	*cmp1△::hisG/cmp1△::hisG*	This study
*cmp1Δ-CaMCA1*	*cnaΔu*	*cmp1△::hisG/cmp1△::hisG::CaMCA1-URA3*	This study
*cmp1Δ-CMP1*	*cnaΔu*	*cmp1△::hisG/cmp1△::hisG::CMP1-URA3*	This study
*cmp1Δ-EXP*	*cnaΔu*	*cmp1△::hisG/cmp1△::hisG::URA3*	This study
*crz1Δ(DSY2195)*	*DSY2188*	*crz1△::hisG/crz1△::hisG-URA3-hisG*	Karababa et al., 2006
*crz1Δu*	*crz1Δ*	*crz1△::hisG/crz1△::hisG*	This study
*crz1Δ-CaMCA1*	*crz1Δu*	*crz1△::hisG/crz1△::hisG::CaMCA1-URA3*	This study
*crz1Δ-CRZ1*	*crz1Δu*	*crz1△::hisG/crz1△::hisG::CRZ1-URA3*	This study
*crz1Δ-EXP*	*crz1Δu*	*crz1△::hisG/crz1△::hisG::URA3*	This study
*camca1Δ*	*CAI4*	*camca1△::hisG/camca1△::hisG-URA3-hisG*	Cao et al., 2009
*CAI4-CaMCA1*	*CAI4*	*ura3△::immm434::CaMCA1-URA3*	This study
*tps1Δ*	*CAI4*	*tps1△::hisG/tps1△::hisG*	Zaragoza et al., 1998
*tps1Δ-EXP*	*tps1Δ*	*tps1△::hisG/tps1△::hisG::URA3*	This study
*tps1Δ-TPS1*	*tps1Δ*	*tps1△::hisG/tps1△::hisG::TPS1-URA3*	This study

**Table 3:** Plasmids used in this study.

plasmid	Parent	Genotype	Reference
pCaEXP	pCaEXP	*C. albicans* expression vector	Care et al., 1999
pCaEXP-MCA1	pCaEXP	expression vector containing *CaMCA1*	Cao et al., 2009
pCaEXP-CMP1	pCaEXP	expression vector containing *CMP1*	This study
pCaEXP-CRZ1	pCaEXP	expression vector containing *CRZ1*	This study
pCaEXP-TPS1	pCaEXP	expression vector containing *TPS1*	This study

### Cell Treatment and Apoptosis Measurement

Yeast cells grown to early exponential phase at 30°C were exposed to different concentrations of H_2_O_2_ for the required time (range 0–3 hours) and then harvested for apoptosis measurement. A terminal deoxynucleotidyltransferase-mediated dUTP-biotin nick end labeling (TUNEL) assay was performed in order to confirm the occurrence of the apoptosis process [4]. *C. albicans* cells were washed twice with PBS and fixed with a solution of 3.6% paraformaldehyde in PBS for 1 hour at 20°C. Cells were rinsed twice with PBS and then incubated with permeabilization solution for 2 minutes on ice. The cells were rinsed in PBS and labeled, using a solution of the label and enzyme solutions from an *in situ* cell death detection kit, fluorescein (Roche Applied Sciences, Mannheim, Germany), with appropriate controls labeled only with the label solution. The cells were incubated for 1 hour at 37°C in a humidified atmosphere in the dark, rinsed in PBS. The staining of the cells was observed by a fluorescence microscopy. Alternatively, the number of cells determined to be positive by the TUNEL assay was quantified using a BD FACSCalibur flow cytometer with excitation and emission wavelength settings at 488 and 520 nm, respectively.

### Assay of the Intracellular Content of Trehalose

For analysis of the intracellular trehalose, the cells grown to early exponential phase at 30°C were exposed to 1 mM H_2_O_2_ for 3 hours. At the indicated times, aliquots of cells (about 5×10^8^) were taken and immediately centrifuged and washed with cold distilled water. Samples were microwaved (700 W) for 3×60 seconds with 30 seconds intervals between each, 1 ml of distilled water was then used to extract the trehalose for 1 hour. After centrifugation at 15,000×g for 10 minutes, the trehalose in the supernatants was analyzed by HPLC-MS with a detection limit of 1 ng. An HPLC system (Agilent1100, Wilmington, Germany) equipped with a G1946 mass spectrometer was used in the analysis. The operating conditions were as follows: Extracts were analyzed after separation of an Agilent Zorbax NH2 Column (4.6 mm×250 mm, 5 mm) at a flow rate of 1.0 ml/min. The mobile phase consisted of methanol∶ water 85∶15 (v/v). The HPLC eluant from the DAD detector was introduced into the mass spectrometer *via* a 1∶3 split. The column temperature was 25°C. A quadrupole mass spectrometer equipped with an ESI interface was used to obtain mass spectra, which were then examined by SIM in negative mode. The nebulizing gas was at 40 psi, and the drying gas temperature was 350°C. The fragmentor was set to 70 V, and the capillary voltage was 3.5 kV. The cell weight was determined as follows: another sample of the same volume of the corresponding cell suspension was filtered through pre-weighed filters (0.22 µm pore size). After washing with PBS, the filters were dried at 37°C for 48 h and then weighed. The trehalose content was showed as nmol/mg.

### Measurement of ROS Levels

Intracellular levels of ROS were measured with DCFH-DA (Molecular Probes, U.S.A.). Briefly, cultured cells were collected by centrifugation and washed three times with PBS. Subsequently, the cells were adjusted to 2×10^7^ cells/ml. After being incubated with 20 µg/ml of DCFH-DA for 30 minutes at 30°C, the cells were exposed to H_2_O_2_ and incubated at 30°C with constant shaking (200 rpm). At specified intervals, cell suspensions were harvested and examined by fluorescence microscope or transferred to the wells of a flat-bottom microplate (BMG Microplate, 96 well, Blank) to detect fluoresence intensity on the POLARstar Galaxy (BMG, Labtech, Offenburg, Germany) with excitation at 485 nm and emission at 520 nm.

### Ca^2+^ Detection

Cells were loaded with 5 µM Fluo-3/AM for 30 minutes at 37°C. Ca^2+^ levels were determined by a fluorescence microscopy. Alternatively, fluorescence intensity values were determined on the POLARstar Galaxy (BMG, Labtech, Offenburg, Germany) with excitation at 488 nm and emission at 525 nm.

### Assessment of Caspase Activity

Caspase activity was detected by staining with D_2_R (CaspSCREEN Flow Cytometric Apoptosis Detection Kit, BioVision, U.S.A.) [10], [11], [41]. According to the manufacturer's instructions, cells were in D_2_R incubation buffer at 30°C for 45 minutes before viewing and counting under a fluorescence microscope with excitation at 488 nm and emission at 530 nm.

### Real-time RT-PCR

RNA isolation and real-time RT-PCR were performed as described previously [42]. The isolated RNA was resuspended in diethyl pyrocarbonate-treated water. The OD_260_ and OD_280_ were measured, and the integrity of the RNA was visualized by subjecting 2 to 5 µl of the samples to electrophoresis through a 1% agarose-MOPS gel. First-strand cDNAs were synthesized from 3 µg of total RNA in a 60 µl reaction volume using the cDNA synthesis kit for RT-PCR (TaKaRa Biotechnology, Dalian, P.R. China) in accordance with the manufacturer's instructions. Triplicate independent quantitative real-time PCR were performed using the LightCycler System (Roche diagnostics, GmbH Mannheim, Germany). SYBR Green I (TaKaRa) was used to visualize and monitor the amplified product in real time according to the manufacturer's protocol. *CaMCA1* was amplified with the forward primer 5′-TATAATAGACCTTCTGGAC-3′ and the reverse primer 5′- TTGGTGGACGAGAATAATG-3′.

The PCR protocol consisted of denaturation program (95°C for 10 seconds), 40 cycles of amplification and quantification program (95°C for 10 seconds, 60°C for 20 seconds, 72°C for 15 seconds with a single fluorescence measurement), melting curve program (60–95°C with a heating rate of 0.1°C per second and a continuous fluorescence measurement) and finally a cooling step to 40°C. A standard curve for each primer set was performed with 1∶10, 1∶25, 1∶50, 1∶100, 1∶250 and 1∶500 dilutions of the cDNAs. The slopes of the standard curves were within 10% of 100% efficiency. The change in fluorescence of SYBR Green I dye in every cycle was monitored by the LightCycler system software, and the threshold cycle (C_T_) above background for each reaction was calculated. The C_T_ value of *ACT1* (amplified with the forward primer 5′-CAACAAGGACAATACAATAG-3′ and the reverse primer 5′- GTTGGTGGACGAGAATAATG -3′) was subtracted from that of the tested genes to obtain a ΔC_T_ value. The ΔC_T_ value of an arbitrary calibrator was subtracted from the ΔC_T_ value of each sample to obtain a ΔΔC_T_ value. The gene expression level relative to the calibrator was expressed as 2^−ΔΔCT^.

## References

[1] Fidel PL (2006). *Candida*-host interactions in HIV disease: relationships in oropharyngeal candidiasis.. Adv Dent Res.

[2] Perlroth J, Choi B, Spellberg B (2007). Nosocomial fungal infections: epidemiology, diagnosis, and treatment.. Med Mycol.

[3] Redding SW, Zellars RC, Kirkpatrick WR, McAtee RK, Caceres MA (1999). Epidemiology of oropharyngeal *Candida* colonization and infection in patients receiving radiation for head and neck cancer.. J Clin Microbiol.

[4] Madeo F, Frohlich E, Frohlich KU (1997). A yeast mutant showing diagnostic markers of early and late apoptosis.. J Cell Biol.

[5] Madeo F, Fröhlich E, Ligr M, Grey M, Sigrist SJ (1999). Oxygen stress: a regulator of apoptosis in yeast.. J Cell Biol.

[6] Madeo F, Herker E, Maldener C, Wissing S, Lachel S (2002). A caspase-related protease regulates apoptosis in yeast.. Mol Cell.

[7] Silva RD, Sotoca R, Johansson B, Ludovico P, Sansonetty F (2005). Hyperosmotic stress induces metacaspase- and mitochondria-dependent apoptosis in *Saccharomyces cerevisiae*.. Mol Microbiol.

[8] Phillips AJ, Sudbery I, Ramsdale M (2003). Apoptosis induced by environmental stresses and amphotericin B in *Candida albicans*.. Proc Natl Acad Sci USA.

[9] Phillips AJ, Crowe JD, Ramsdale M (2006). Ras pathway signaling accelerates programmed cell death in the pathogenic fungus *Candida albicans*.. Proc Natl Acad Sci USA.

[10] Al-Dhaheri RS, Douglas LJ (2010). Apoptosis in Candida biofilms exposed to amphotericin B.. J Med Microbiol.

[11] Cao YY, Huang S, Dai BD, Zhu ZY, Lu H (2009). *Candida albicans* cells lacking CaMCA1-encoded metacaspase show resistance to oxidative stress-induced death and change in energy metabolism.. Fungal Genet Biol.

[12] Arguelles JC (2000). Physiological roles of trehalose in bacteria and yeast: a comparative analysis.. Arch Microbiol.

[13] Richards AB, Krakowka S, Dexter LB, Schmid H, Wolterbeek APM (2002). Trehalose: a review of properties, history of use and human tolerance, and results of multiple safety studies.. Food Chem Toxicol.

[14] Elbein AD, Pan YT, Pastuszak I, Carroll D (2003). New insights on trehalose: a multifunctional molecule.. Glycobiology.

[15] Van Dijck P, DeRop L, Szlufcik K, VanAel E, Thevelein JM (2002). Disruption of the *Candida albicans TPS2* gene encoding trehalose-6-phosphate phosphatase decreases infectivity without affecting hypha formation.. Infect Immun.

[16] Zaragoza O, de Virgilio C, Ponton J, Gancedo C (2002). Disruptionin *Candida albicans* of the *TPS2* gene encoding trehalose-6-phosphate phosphatase affects cell integrity and decreases infectivity.. Microbiology.

[17] Thevelein JM, Hohmann S (1995). Trehalose synthase: guard to the gate of glycolysis in yeast?. Trends Biochem Sci.

[18] Zaragoza O, Blazquez MA, Gancedo C (1998). Disruption of the *Candida albicans TPS1* gene encoding trehalose-6P- synthase impairs formation of hyphae and decreases infectivity.. J Bacteriol.

[19] Alvarez P, Francisco J, Zaragoza O, Pedreno Y, Arguelles JC (2002). Protective role of trehalose during severe oxidative stress caused by hydrogen peroxide and the adaptive oxidative stress response in *Candida albicans*.. Microbiology.

[20] Bader T, Schroppel K, Bentink S, Agabian N, Kohler G (2006). Role of calcineurin in stress resistance, morphogenesis, and virulence of a *Candida albicans* wild-type strain.. Infect Immun.

[21] Cannon RD, Lamping E, Holmes AR, Niimi K, Tanabe K (2007). *Candida albicans* drug resistance: another way to cope with stress.. Microbiology.

[22] Hemenway CS, Heitman J (1999). Calcineurin. Structure, function and inhibition. Cell.. Biochem Biophys.

[23] Steinbach WJ, Reedy JL, Crame RA, Perfect JR, Heitman J (2000). Calcineurin: Form and function.. Physiol Rev.

[24] Pozniakovsky AI, Knorre DA, Markova OV, Hyman AA, Skulachev VP (2005). Role of mitochondria in the pheromone- and amiodarone-induced programmed death of yeast.. J Cell Biology.

[25] Gupta SS, Ton VK, Beaudr V, Rulli S, Cunningham K (2003). Antifungal activity of amiodarone is mediated by disruption of calcium homeostasis.. J Biol Chem.

[26] Karababa M, Valentino E, Pardini G, Coste AT, Bille J (2006). CRZ1, a target of the calcineurin pathway in *Candida albicans.*. Mol Microbiol.

[27] Onyewu C, Wormley FL, Perfec JR, Heitman J (2004). The calcineurin target, Crz1, functions in azole tolerance but is not required for virulence of *Candida albicans*.. Infect Immun.

[28] Sanglard D, Ischer F, Marchetti O, Entenza J, Bille J (2003). Calcineurin A of *Candida albicans*: Involvement in antifungal tolerance, cell morphogenesis and virulence.. Mol Microbiol.

[29] Stathopoulos AM, Cyert MS (1997). Calcineurin acts through the *CRZ1/TCN1*-encoded transcription factor to regulate gene expression in yeast.. Genes Dev.

[30] Vachova L, Palkova Z (2005). Physiological regulation of yeast cell death in multicellular colonies is triggered by ammonia.. J Cell Biol.

[31] Martínez-Esparza M, Aguinaga A, González-Párraga P, García-Peñarrubia P, Jouault T (2007). Role of trehalose in resistance to macrophage killing: study with a *tps*1/*tps*1 trehalose-deficient mutant of *Candida albicans*.. Clin Microbiol Infect.

[32] Liu Q, Xu L, Jiao SX, Wang TX, Song Y (2009). Trehalose inhibited the the phagocytosis of refrigerated platelets in vitro via preventing apoptosis.. Transfusion.

[33] Chen W, Zhang X, Liu M, Zhang J, Ye Y (2009). Trehalose protects against ocular surface disorders in experimental murine dry eye through suppression of apoptosis.. Exp Eye Res.

[34] Khan MA, Chock PB, Stadtman ER (2005). Knockout of caspase-like gene, *YCA1*, abrogates apoptosis and elevates oxidized proteins in *Saccharomyces cerevisiae*.. Proc Natl Acad Sci USA.

[35] Madeo F, Herker E, Wissing S, Jungwirth H, Eisenberg T (2004). Apoptosis in yeast.. Curr Opin Microbiol.

[36] Wadskog I, Maldener C, Proksch A, Madeo F, Adler L (2004). Yeast lacking the *SRO7/SOP1*-encoded tumor suppressor homologue show increased susceptibility to apoptosis-like cell death on exposure to NaCl stress.. Mol Biol Cell.

[37] Bozhkov PV, Suarez MF, Filonova LH, Daniel G, Zamyatnin AA (2005). Cysteine protease mcII-Pa executes programmed cell death during plant organogenes*is.*. Proc Natl Acad Sci USA.

[38] Hoeberichts FA, ten Have A, Woltering EJ (2003). A tomato metacaspase gene is upregulated during programmed cell death in *Botrytis* cinerea-infected leaves.. Planta.

[39] Suarez MF, Filonova LH, Smertenko A, Savenkov EI, Clapham DH (2004). Metacaspase-dependent programmed cell death is essential for plant embryogenesis.. Curr Biol.

[40] Watanabe N, Lam E (2005). Two *Arabidopsis* metacaspases *AtMCP1b* and *AtMCP2b* are arginine/lysine-specific cysteine proteases and activate apoptosis-like cell death in yeast.. J Biol Chem.

[41] Care RS, Trevethick J, Binley KM, Sudbery PE (1999). The *MET* promoter: a new tool for *Candida albicans* molecular genetics.. Mol Microbiol.

[42] Wang Y, Cao YY, Jia XM, Cao YB, Gao PH (2006). Cap1p is involved in multiple pathways of oxidative stress response in *Candida albicans*.. Free Radical Biol Med.

